# The CDE region of feline Calicivirus VP1 protein is a potential candidate subunit vaccine

**DOI:** 10.1186/s12917-024-03914-2

**Published:** 2024-03-05

**Authors:** Lisha Li, Zirui Liu, Jiale Shi, Mengfang Yang, Yuanyuan Yan, Yanan Fu, Zhou Shen, Guiqing Peng

**Affiliations:** 1 State Key Laboratory of Agricultural Microbiology, College of Veterinary Medicine, Huazhong Agricultural University, Wuhan, 430070 China; 2 Key Laboratory of Preventive Veterinary Medicine in Hubei Province, The Cooperative Innovation Center for Sustainable Pig Production, Wuhan, 430070 China

**Keywords:** FCV, CDE, IgG, IgA, Neutralizing antibody, TNF-α

## Abstract

**Background:**

Feline calicivirus (FCV) infection causes severe upper respiratory disease in cats, but there are no effective vaccines available for preventing FCV infection. Subunit vaccines have the advantages of safety, low cost and excellent immunogenicity, but no FCV subunit vaccine is currently available. The CDE protein is the dominant neutralizing epitope region of the main antigenic structural protein of FCV, VP1. Therefore, this study evaluated the effectiveness of the CDE region as a truncated FCV VP1 protein in preventing FCV infection to provide a strategy for developing potential FCV subunit vaccines.

**Results:**

Through the prediction of FCV VP1 epitopes, we found that the E region is the dominant neutralizing epitope region. By analysing the spatial structure of VP1 protein, 13 amino acid sites in the CD and E regions were found to form hydrogen bonding interactions. The results show the presence of these interaction forces supports the E region, helping improve the stability and expression level of the soluble E protein. Therefore, we selected the CDE protein as the immunogen for the immunization of felines. After immunization with the CDE protein, we found significant stimulation of IgG, IgA and neutralizing antibody production in serum and swab samples, and the cytokine TNF-α levels and the numbers of CD4+ T lymphocytes were increased. Moreover, a viral challenge trial indicated that the protection generated by the CDE subunit vaccine significantly reduced the incidence of disease in animals.

**Conclusions:**

For the first time, we studied the efficacy of the CDE protein, which is the dominant neutralizing epitope region of the FCV VP1 protein, in preventing FCV infection. We revealed that the CDE protein can significantly activate humoral, mucosal and cellular immunity, and the resulting protective effect can significantly reduce the incidence of animal disease. The CDE region of the FCV capsid is easy to produce and has high stability and excellent immunogenicity, which makes it a candidate for low-cost vaccines.

**Supplementary Information:**

The online version contains supplementary material available at 10.1186/s12917-024-03914-2.

## Background

Feline calicivirus (FCV) mainly infects young cats and has a high incidence. Infected cats can experience fever, oral ulcers, chronic stomatitis, conjunctivitis, rhinitis and pneumonia [1]. In addition, virulent, systemic FCV (VS-FCV) strains that cause severe systemic diseases with a high mortality rate have been reported in cats in recent years [2]. Moreover, there is no specific drug available for treating FCV.

**Fig. 1:**
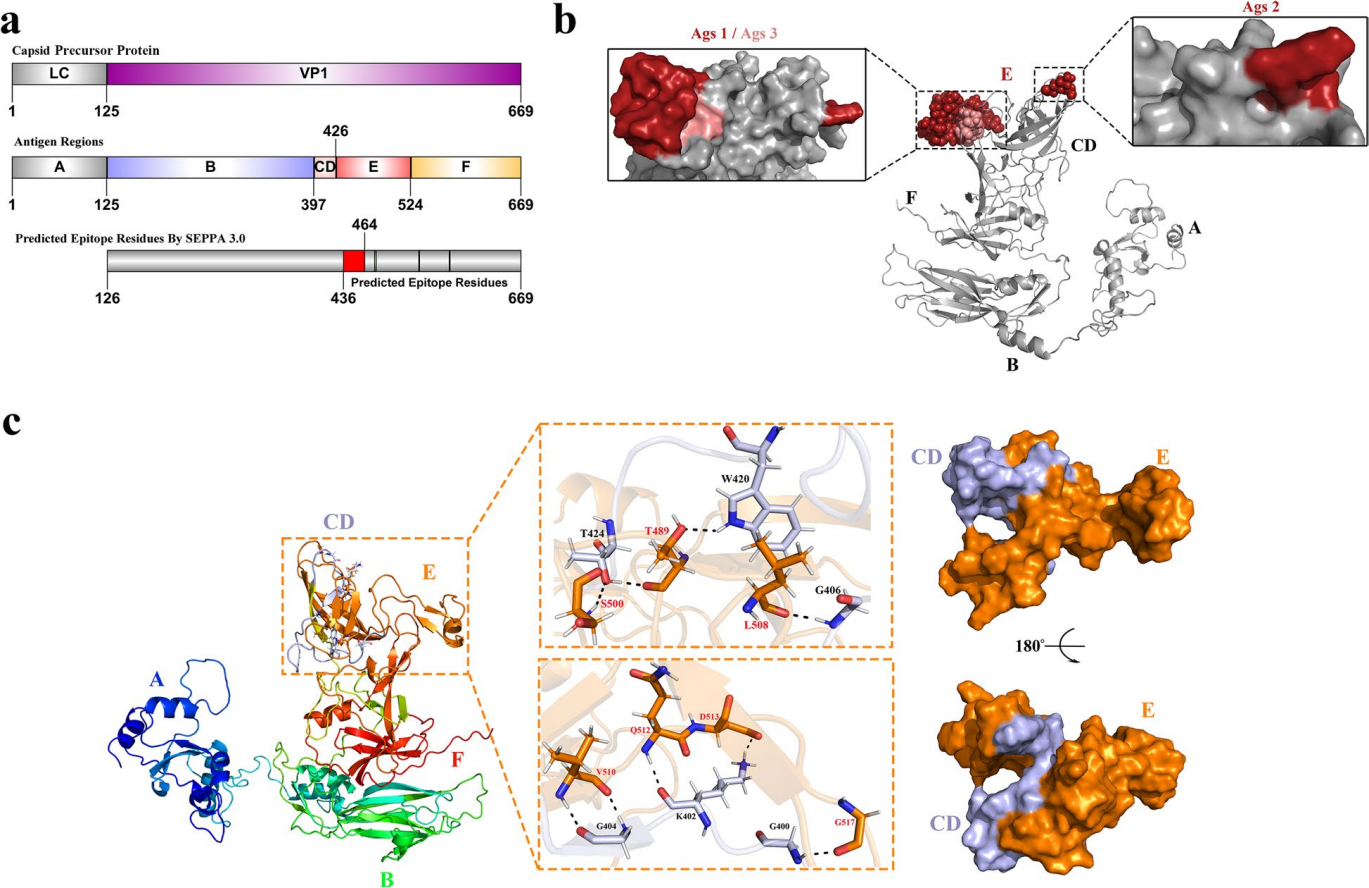
Schematic representation, epitope prediction and structural analysis of FCV Pre-VP1. **a** Schematic representation of FCV Pre-VP1, which was cleaved into the mature proteins LC and VP1. Pre-VP1 was divided into antigenic regions (A–F), and a linear diagram of the predicted epitopes of FCV Pre-VP1 was constructed with SEPPA 3.0. **b** Potential epitopes of FCV Pre-VP1 predicted by SEPPA 3.0 were shown (threshold > 0.19; the E and F regions were shown in red and pink, respectively). **c** Structure of FCV Pre-VP1 and analysis of amino acid interaction sites between the CD and E regions. (the CD and E regions are represented by grey and orange, respectively)

FCV is a small, nonenveloped RNA virus that has a linear, single positive-strand genome of 7.7 kb. This virus belongs to the genus Vesuvius of the family Caliciviridae [3] and contains three functional open reading frames (ORFs). ORF1 at the 5′ end of the genome encodes the 200-kDa polyprotein NS1, which is processed by the 3C-like cysteine proteinase into six mature nonstructural proteins, p5.6, p32, p39, p30, VPg and Pro-pol [4]. ORF2 encodes the 73-kDa capsid precursor protein (Pre-VP1). Notably, Pre-VP1 is cleaved by protease after translation into the leader capsid protein (LC, 14 kDa) and the mature capsid protein (VP1, 60 kDa) (Fig. 1a) [5]. VP1 is the main antigenic structural protein that can self-assemble into virus-like particles (VLPs) [4, 6]. ORF3 encodes the structural protein VP2 (12.2 kDa) at the 3′ end of the genome, which is essential for the production of infectious virions [7]. Pre-VP1 can be divided into six regions, A to F regions, according to the amino acid sequence and antigen region (Fig. 1a, b) [8]. Region A (aa 1- aa 125) is located at the amino terminus and is cut into the LC during VP1 maturation. Region B (aa 126-aa 397) is highly conserved and is the core structure of the ATP/GTP-binding site, and region C (aa 398-aa 401) is a short highly variable sequence. Region D (aa 402-aa 426) is highly conserved. The E region (aa 427-aa 524) is highly variable and further divided into 5′ and 3′ highly variable regions (HVRs), which are separated by a conserved central domain [9]. Region E of VP1 is responsible for interaction with the cell receptor feline junctional adhesion molecule 1 (FeJAM-1) [10] and may contain neutralizing epitopes of VP1 [8, 9, 11–13]. The F region (aa 525-aa 668) is a highly conserved carboxyl terminus.

Vaccination is usually the most effective way to prevent FCV infection. Currently, inactivated and attenuated vaccines are often used for FCV prevention. Inactivated vaccines (IV) stimulate the production of neutralizing antibodies (NAbs) by responding to specific antigens outside the cell (humoral immunity). Attenuated vaccines stimulate cellular immunity of the immune system by mimicking natural infections in addition to humoral responses (cell-mediated immunity). Despite widespread vaccination with the attenuated F9 strain and IV, FCV-related illness continues to occur in healthy cats [14, 15]. Although immunization with the FCV vaccine prevents disease, it does not prevent infection [16–18]. Furthermore, inactivated and attenuated vaccines exhibit incomplete inactivation [19], reemergence of virulence [20] and other security issues. In contrast, subunit vaccines have high yields, are convenient to use and are safe. In addition, subunit vaccines mixed with appropriate adjuvants may simultaneously stimulate humoral, cellular and mucosal immunity [21, 22]. However, there is currently no FCV protein-based vaccine available for use.

VP1, as the main antigenic structural protein of FCV, is often used to design FCV subunit vaccines. Several studies have reported that VLPs assembled from the VP1 protein expressed by a baculovirus expression system [4, 6] can produce FCV-specific NAbs [4]. However, the cost of baculovirus expression systems is an important challenge [23–25]. According to previous reports, the presence of many neutralizing epitopes in the E region has been predicted. Four linear epitopes and two conformational epitopes are found in the E region of the VP1 protein and are located in the aa 426 ~ aa 460 and aa 490 ~ aa 520, respectively [13]. The highly variable region E of the FCV VP1 gene contains major linear B-cell epitopes (BCEs) and neutralizing epitopes [11, 26]. However, although the E region represents a dominant epitope, the immunogenicity of the E region and its role in preventing FCV infection have not been reported.

In past studies, serological IgG and NAbs have generally been considered markers for evaluating the effectiveness of FCV vaccination [27]. However, with the use of vaccines in recent years, there is not always a strong correlation between the occurrence or prevention of disease and various detection indicators. A study of bivalent IV found no association between NAbs titres and clinical protection in some individuals, although high antibody titres predicted clinical protection [28]. Some immunized cats are protected from FCV infection despite inadequate IgG and NAbs [16, 27–30]. These results may not be entirely surprising. In fact, circulating antibodies have limited protective effects against mucosal viruses. It may be interesting if the secretory IgA (sIgA) and NAbs levels in the mucosa can be accurately measured [29]. These studies have shown that indicators for evaluating the effectiveness of FCV vaccination should include not only humoral immunity (IgG, NAbs) but also cellular [16] and mucosal immunity [27, 29]. Therefore, we hypothesize that in addition to humoral immunity, cellular and mucosal immunity play a role in the prevention of and protection against FCV infection.

To study the effectiveness of the subunit vaccine against FCV, rural felines were immunized with a truncated VP1 protein comprising the CDE region containing the FCV neutralizing epitope as the immunogen. The immune effect of the FCV CDE protein was comprehensively analysed in terms of humoral, cellular and mucosal immunity to explore the immunogenicity and effectiveness of the CDE protein as a candidate vaccine.

## Results

### Epitope prediction and structural analysis of the pre-VP1 protein

A linear and structural diagram of the FCV VP1 protein is presented. To accurately predict the epitope of VP1, we conducted whole-genome sequencing of FCV strain W, which was isolated and identified in our laboratory. The nucleotide sequence and ORF sequence of VP1 were obtained and uploaded to the website AlphaFold2 for protein structure prediction. A PDB file containing the VP1 protein structure was uploaded to the SEPPA3.0 website for epitope prediction [31]. With 0.19 as the threshold for analysis, three dominant regions were ultimately screened (Fig. 1b, red and pink regions). All three areas are on the surface. After analysis, it was found that the three predicted epitopes were mostly located in the E region (aa 436 to aa 464 and aa 477 to aa 479), named antigenic sites 1 (ags1) and ags2, respectively (shown in red). Four amino acid residues were located in the F region (Ile535, Gly536, Tyr575, and Val576, named ags3 and are shown in pink) (Fig. 1b and Table 1). As previously reported, the dominant epitopes of the FCV VP1 protein were concentrated in the E region.

**Table 1:** SEPPA3.0 prediction results (score > 0.19)

Chain ID	ResSeq	ResName	score	Location
A	436	ALA	0.345	Surface
A	438	THR	0.576	Surface
A	439	ASN	0.578	Surface
A	440	SER	0.602	Surface
A	441	SER	0.638	Surface
A	442	GLY	0.611	Surface
A	443	SER	0.666	Surface
A	444	ASP	0.616	Surface
A	445	ILE	0.518	Surface
A	446	ALA	0.366	Surface
A	447	THR	0.402	Surface
A	448	ALA	0.297	Surface
A	449	SER	0.304	Surface
A	450	ALA	0.452	Surface
A	451	TYR	0.318	Surface
A	452	ASP	0.272	Surface
A	453	THR	0.356	Surface
A	454	ALA	0.439	Surface
A	455	ASP	0.476	Surface
A	456	VAL	0.587	Surface
A	457	ILE	0.532	Surface
A	458	LYS	0.554	Surface
A	459	ASN	0.403	Surface
A	460	ASN	0.445	Surface
A	464	ARG	0.223	Surface
A	477	GLY	0.218	Surface
A	479	LYS	0.194	Surface
A	535	ILE	0.277	Surface
A	536	GLY	0.288	Surface
A	575	TYR	0.289	Surface
A	576	VAL	0.216	Surface

By analysing the predicted structure of the FCV strain W VP1 protein, we found the spatial distance between the CD and E regions was very close. After analysing the interaction between the CD and E regions, we found hydrogen bonding forces between the 6 amino acids in the CD region (Gly400, Lys402, Gly404, Gly406, Trp420, and Thr424) and the 7 amino acids in the E region (Gly517, Gln512, Asp513, Val510, Leu508, Thr489, and Ser500) (Fig. 1c). After the surface is displayed, the compact spatial position of the CD and E regions becomes more obvious. We hypothesize that the hydrogen bond interaction between these sites contributes to the conformational stability and increased expression level of the E protein. This conjecture was confirmed in subsequent experiments. The expression level and stability of the E protein alone were far lower than those of the CDE fusion protein.

Therefore, in subsequent experiments, we used a prokaryotic expression system to express the CDE protein of FCV strain W as a vaccine antigen and thus verify the immunogenicity of the E region.

### Preparation of the CDE protein as a vaccine

**Fig. 2:**
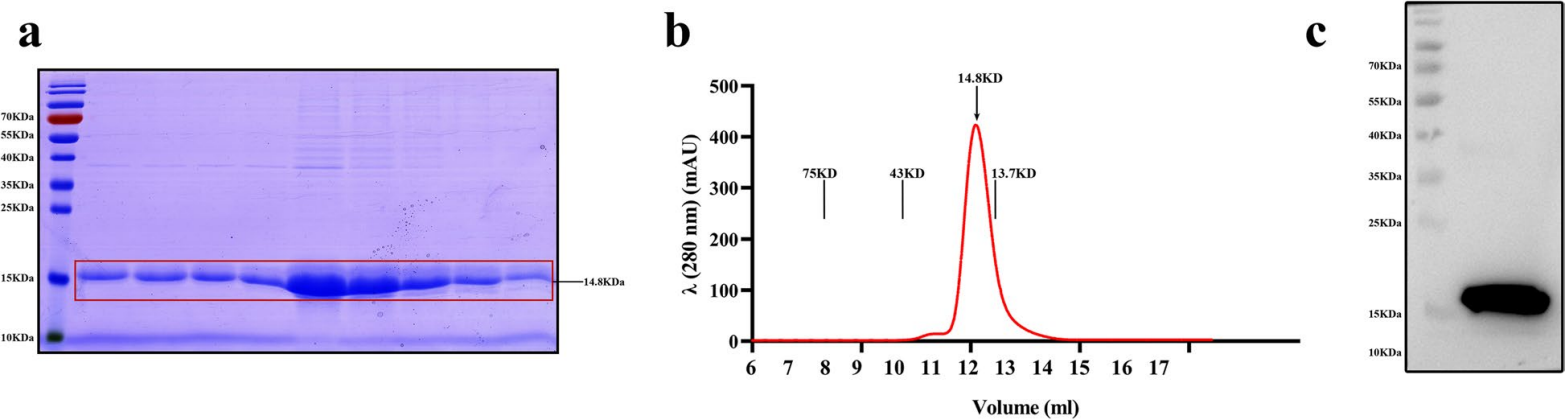
Prokaryotic expression and purification of CDE protein. **a** The expression of recombinant CDE protein was assessed by SDS–PAGE analysis. This image has been cropped, and the original gel is presented in additional file 1. **b** SEC assay of CDE protein with markers at75-, 43- and 13.7-kDa. **c** Antigenicity was verified using HRP-conjugated 6*His Tag mouse antibody. This image has been cropped, and the full-length blot is presented in additional file 2

The CDE gene was cloned for protein expression in the pET42b plasmid with an 8*His tag. PCR was used to verify the constructed recombinant expression plasmid pET42b-CDE (NdeI/XhoI). The sizes of target bands in the electrophoretograms were as expected, and the sequencing results indicated that the recombinant expression plasmids exhibited no mutations. The pET42b-CDE vector was subsequently transformed into *E. coli* strain BL21. The supernatant of the crushed bacterial solution was eluted by nickel column affinity chromatography, and the expression of the CDE protein was observed by SDS–PAGE analysis, which revealed an obvious single target band at 14.8 kDa (Fig. 2a). Size-exclusion chromatography (SEC) was used to further purify the CDE protein to improve the safety of the subunit vaccine. The SEC data showed that the CDE protein had a peak at the corresponding location. The results indicated that the CDE protein was present in a single and pure state in solution. Each standard protein marker is shown at different positions of 75, 43, and 13.7 kDa (Fig. 2b). The western blot results showed that the recombinant CDE protein was expressed in the supernatant of the BL21-pET42b-CDE strains. The specific band at 14.8 kDa in Fig. 2c shows that the recombinant CDE protein was successfully harvested.

### IgG, IgA and neutralizing antibody responses in serum and swabs after immunization

**Fig. 3:**
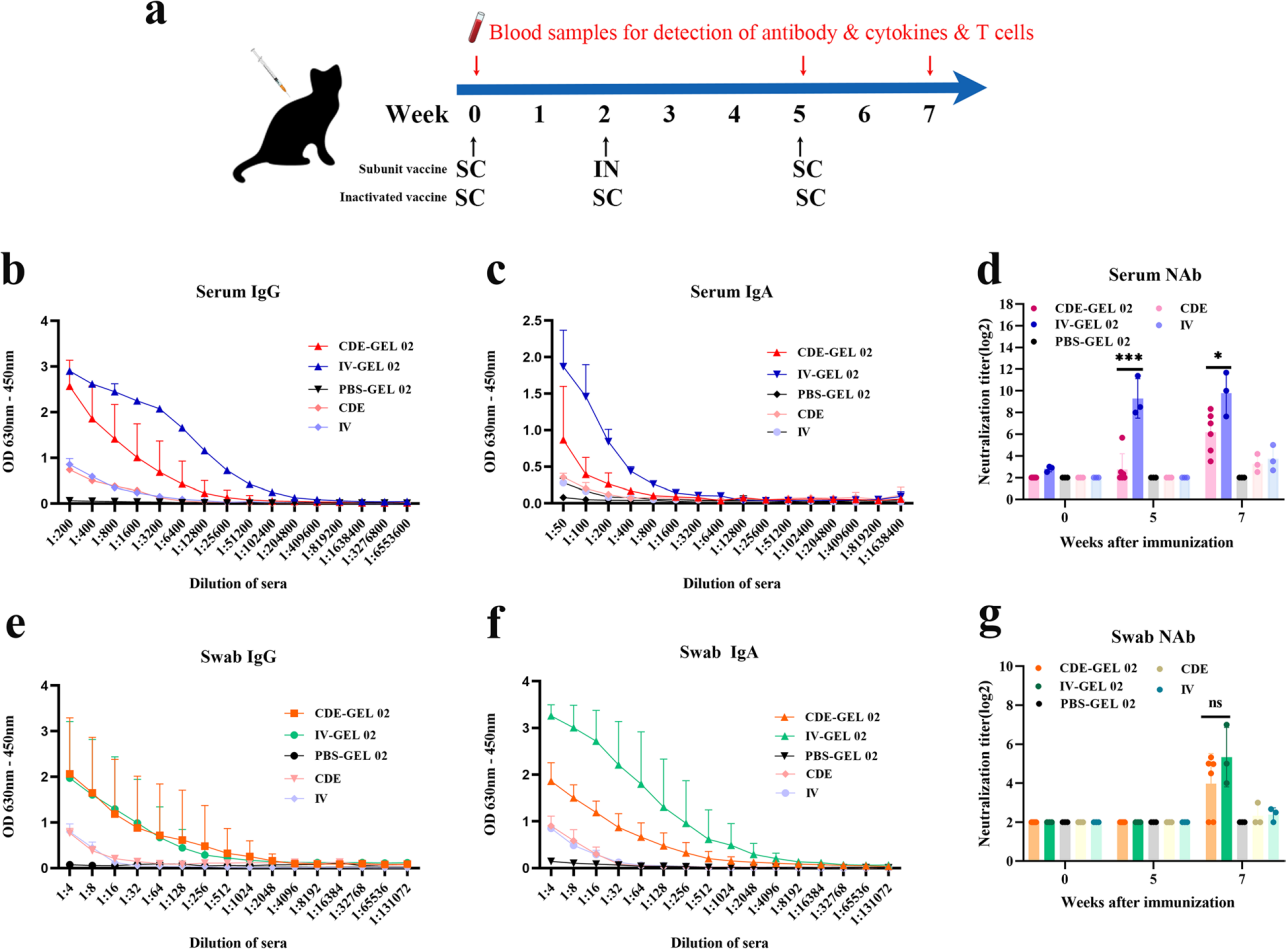
The IgG, IgA and neutralizing antibody levels in serum and ENM swabs were detected. **a** Diagram of the immunization program. At 0, 2 and 5 weeks, the felines were immunized via subcutaneous and nasal drops. Serum and ENM samples were collected at 0, 5 and 7 weeks. **b**, (**c**), (**e**), and (**f**) 7 weeks after immunization, the levels of IgG and IgA antibodies in serum and swabs were detected by ELISA. **d**, (**g**) At 0, 5, and 7 weeks after immunization, the levels of NAbs in serum and swabs were detected via virus neutralization tests. ^*^p < 0.05, ^*^^*^^*^p < 0.001, and ns indicates no significant difference

To evaluate the specific immunogenicity of the generated CDE vaccine candidates, felines without specific pathogens were selected and divided into 5 groups: CDE-GEL 02, IV-GEL 02, PBS-GEL 02, CDE and IV groups. Three immunizations were performed at 0 W, 2 W, and 5 W (Fig. 3a). The levels of IgG and IgA in the serum and eye, nose and mouth (ENM) swab samples were subsequently measured by ELISA after 7 weeks. The results revealed higher levels of IgG and IgA antibodies in serum and swabs at 7 weeks after immunization with CDE-GEL 02 than in the PBS group (Fig. 3b, c, e, f). The OD value of IgG in serum and swabs and that of IgA in swab reached 2 (Fig. 3b, e, f), and the serum IgA value ranged from 1 to 1.5 (Fig. 3c). This finding suggests that with the help of adjuvants, the CDE protein can effectively stimulate and induce humoral and mucosal immunity. In addition, we found that the levels of IgG and IgA produced in the IV-GEL 02 group were mostly higher than those in the CDE-GEL 02 group. However, similar levels of IgG were produced in the swab samples with the two vaccines (Fig. 3e). The CDE and IV groups produced particularly trace levels of IgG and IgA in the absence of the adjuvant GEL 02, which also illustrates the importance of adjuvants for subunit and inactivated vaccines.

Meanwhile, the NAbs levels in serum and swab samples were measured. NAbs production was detected in serum at week 5 after CDE GEL 02 immunization. The serum antibody level increased further, reaching a level of 2^7^ (the sample dilution was 1: 128) at week 7 (Fig. 3d). However, NAbs were not detected in the swab samples until week 7, at which point the NAbs concentration reached 2^4^ (1: 16; Fig. 3g). Therefore, the CDE protein can stimulate both humoral and mucosal immunity. Moreover, 5 weeks after immunization with IV-GEL 02, NAbs were detected in serum at a high level of 2^9^ (1: 512). The NAbs levels in swab samples were also detected at 7 weeks after immunization with IV-GEL 02, reaching a level of 2^5^ (1: 32), and no significant difference was found between the CDE-GEL 02 group and the IV-GEL 02 group. In the PBS-GEL 02, CDE and IV groups, only trace amounts of NAbs were detected throughout the process.

### Determination of TNF-α, CD4+ and CD8+ T lymphocytes levels after immunization

**Fig. 4:**
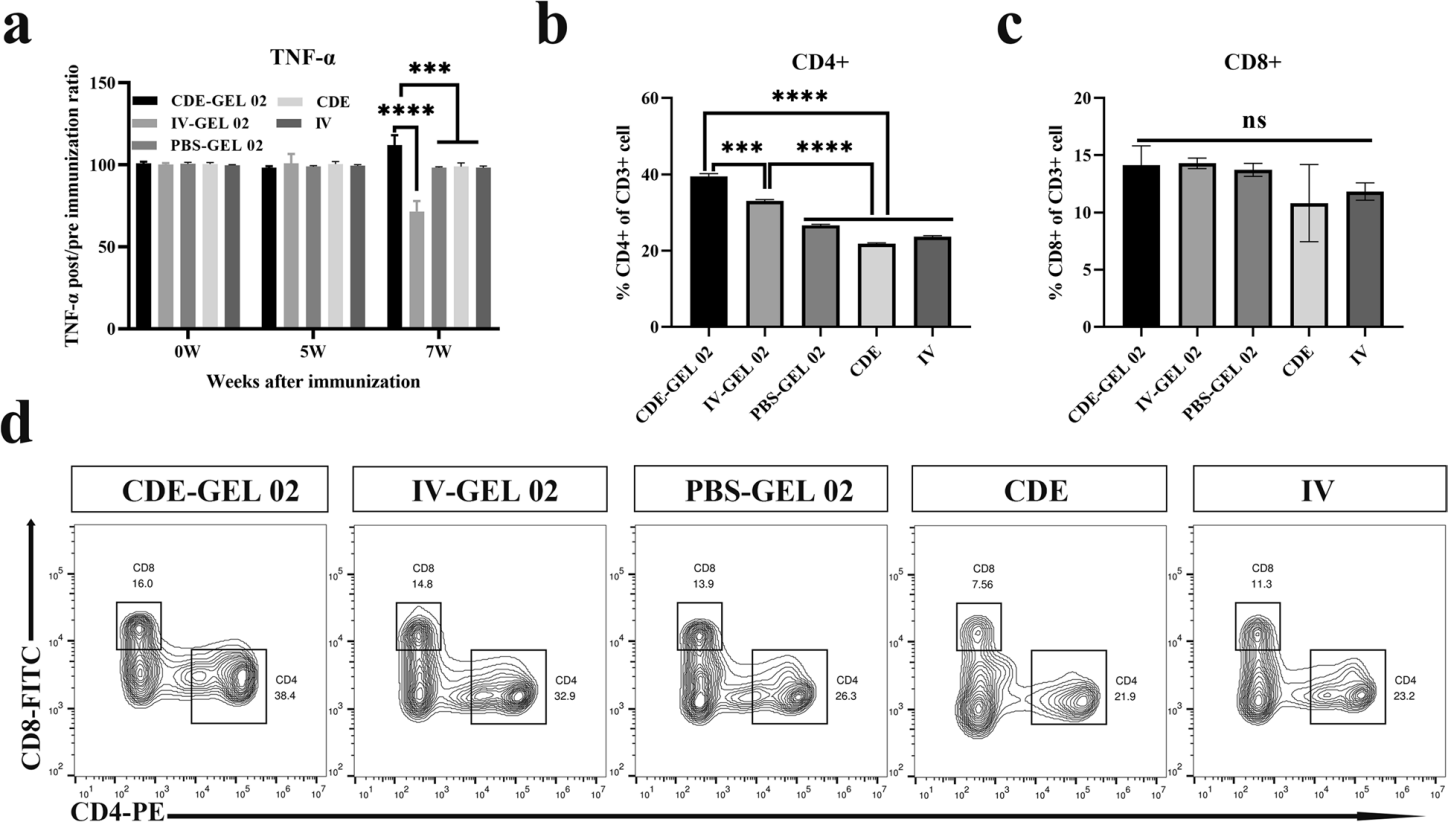
Detection of TNF-α, CD4+ and CD8+ T lymphocytes. **a** Detection of TNF-α levels in serum after immunization. **b**, (**c**) Determination of the proportion of CD4+, CD8+ T lymphocytes among blood at 7 weeks after immunization. **d** Contour plot of the CD4+ and CD8+ T lymphocytes at 7 weeks after immunization in each group. The bars represent the means ± standard deviations of three independent experiments. ^*^^*^^*^ *p* < 0.001, *** *p* < 0.0001, and ns indicates no significant difference

We subsequently studied the cellular immunity induced by CDE-GEL 02 immunization. The TNF-α concentration was assessed using commercial ELISA kits. Results demonstrated the level of TNF-α at 7 weeks after immunization with CDE-GEL 02 was higher than that before immunization (Fig. 4a). However, a significant decreasing trend was observed 7 weeks after IV-GEL 02 immunization compared with before immunization. In the PBS-GEL 02, CDE and IV groups, the changes in TNF-α expression were almost undetectable.

Additionally, the proportions of CD4+ and CD8+ T lymphocytes among the total CD3+ T lymphocytes in each group were measured at 7 weeks postimmunization by flow cytometry. The results showed that the proportion of CD4+ T lymphocytes was significantly increased in the CDE-GEL 02 group (Fig. 4b, d), indicating expansion of the CD4+ T lymphocytes population. This finding is consistent with the detected increase in the TNF-α levels. Unexpectedly, the IV-GEL 02 group also exhibited a slight increase in the proportion of CD4+ T lymphocytes. These results indicated that both CDE-GEL 02 and IV-GEL 02 could induce a certain CD4+ T lymphocytes level. No significant difference in the proportion of CD8+ T lymphocytes was detected among the immunization groups (Fig. 4c, d).

### Protective efficacy of the CDE protein

**Fig. 5:**
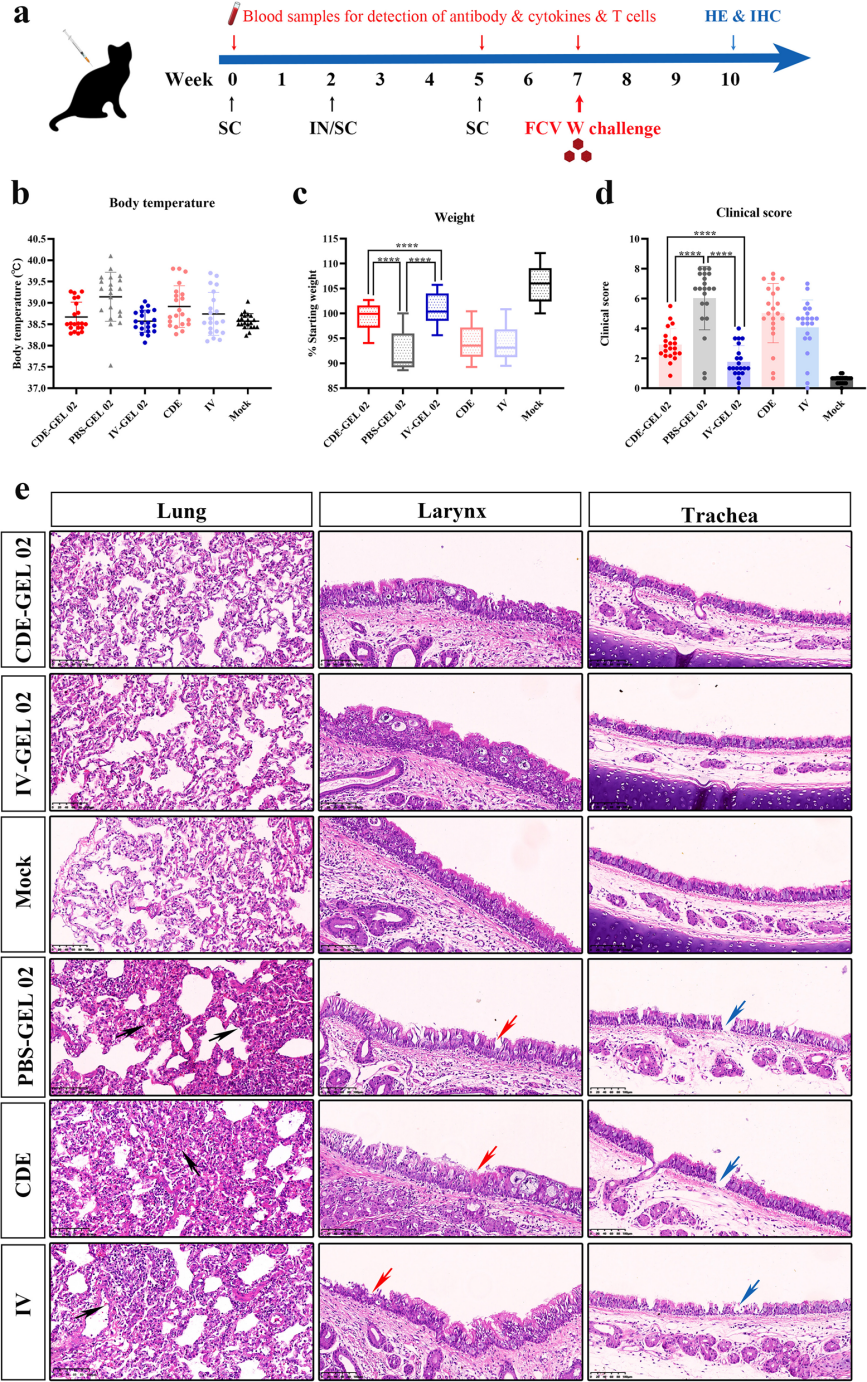
Protective efficacy of the CDE protein against challenge after immunization. **a** Diagram of the FCV-W challenge. The body temperature (**b**), body weight (**c**) and clinical score (**d**) data of all groups were monitored after viral challenge. **e** Histopathological images of the lung, larynx and trachea of the inoculated felines on day 21 post infection. **** *p* < 0.0001

At 7 weeks after immunization, the protective efficacy of each group was evaluated. Felines infected with FCV strain W exhibit symptoms such as conjunctivitis, increased nasal discharge, sneezing, and difficulty breathing. A homologous challenge study was performed by direct nose drop inoculation of 10^7^ TCID_50_ of FCV strain W into the felines (Fig. 5a). A mock group was used as a blank control without virus infection and behaved normally throughout the experiment. In the PBS, CDE and IV groups showed typical FCV symptoms such as fever (≥39.5 °C), significant weight loss, loss of appetite, depression, increased eye secretions, conjunctivitis, and nasal secretions changed from serous to suppurative. In contrast, the CDE-GEL 02-immunized group exhibited a significantly reduced degree of FCV disease onset within 21 days after viral challenge. The body temperature of the CDE-GEL 02 group fluctuated within the normal range after challenge, and no fever was recorded (Fig. 5b). 3/6 animals exhibited transient loss of appetite and weight loss in the CDE-GEL 02 group (Fig. 5c). In addition, 2/6 of the cats exhibited slight discharge from the eye (Fig. 5d), which lasted 6–8 days. By the end of the post challenge surveillance period, the CDE-GEL 02 group had almost returned to normal. A similar phenomenon was observed in the IV-GEL 02 group. The IV-GEL 02 group did not exhibit fever after challenge, but a slight loss of appetite and weight loss were observed. 1/3 of the animal developed mild discharge from the eyes and nose but returned to normal 3–5 days after onset of the disease. These results illustrate that the CDE-GEL 02 and IV-GEL 02 groups showed significant improvements in the weight change rate and FCV clinical score compared with the PBS-GEL 02 group.

One feline of each group was euthanized on the 21st day after challenge to observe the pathological changes in the lung, larynx and trachea. As expected, the PBS-GEL 02, CDE, and IV groups exhibited severe interstitial pneumonia (Fig. 5e) with thickened alveolar walls. The lungs exhibited congestion, bleeding (shown by black arrows), a small amount of fibrin exudation from the alveolar lumen, and shedding of alveolar epithelial cells. Slight alveolar wall thickening was observed in the CDE-GEL 02-immunized group, but no bleeding or inflammatory cell infiltration was observed. The IV-GEL 02 group exhibited a similar pattern. Although some animals did not show any symptoms at the end of the monitoring period, it is possible that the early stages of the disease caused some subtle damage to the lungs. The larynx and trachea of the feline in the PBS-GEL 02, CDE, and IV groups exhibited different degrees of mucosal layer cell shedding and necrosis (shown by red and blue arrows, respectively). The larynx and trachea mucosa of the CDE-GEL 02 and IV-GEL 02 groups were intact and smooth, and no obvious cell shedding was observed.

**Fig. 6:**
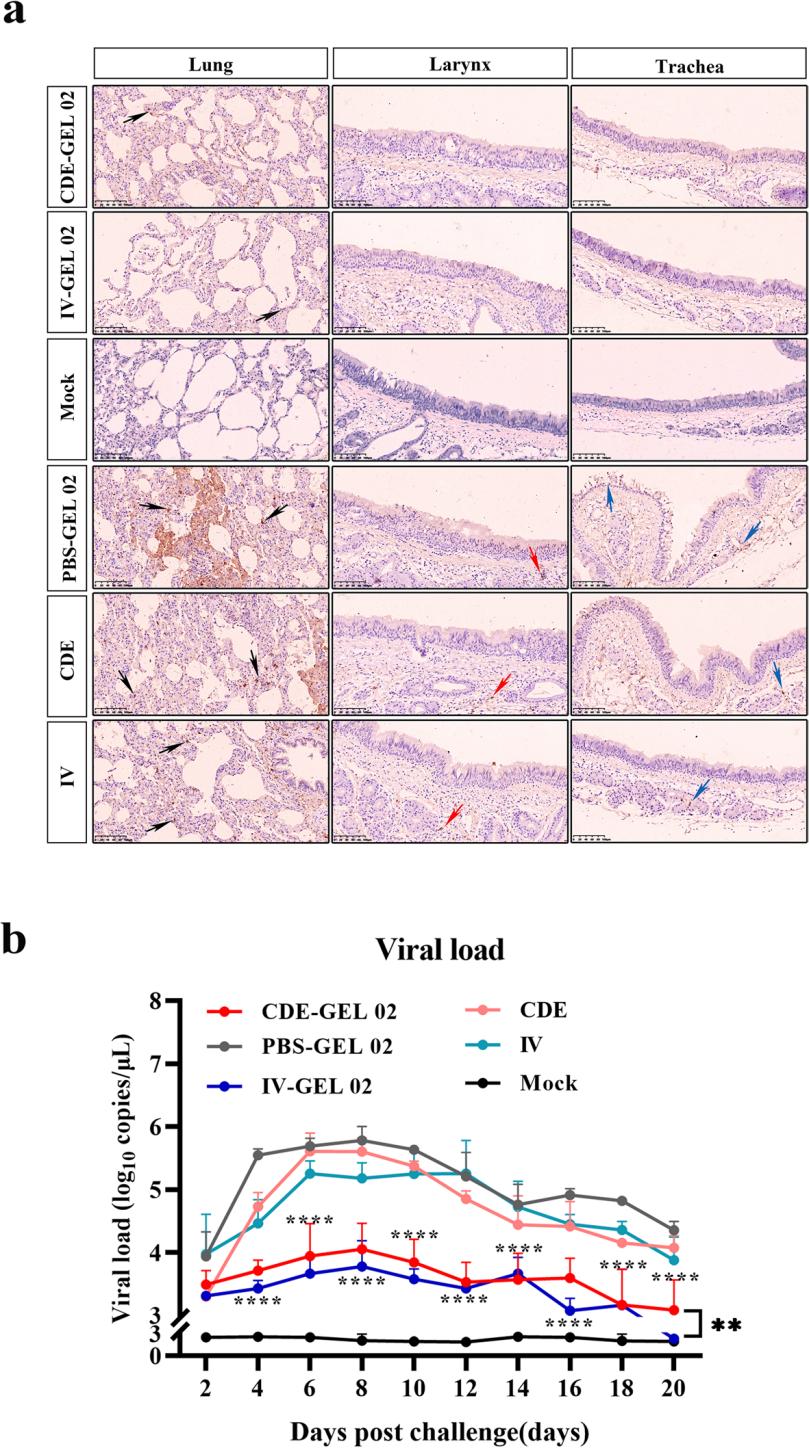
Immunohistochemical of the lung, larynx, trachea and viral load in ENM swab samples. **a** Immunohistochemical images of the lung, larynx and trachea on day 21 post infection. **b** Viral genomic RNA load testing of all groups based on ENM swab samples after viral challenge. ^*^^*^ *p* < 0.01, ^*^^*^^*^ *p* < 0.0001

On the other hand, immunohistochemical techniques were used to detect FCV antigen localization in lung, larynx and trachea tissues. The results showed a large concentration of brown–yellow positive signals (indicated by the black arrow) and alveolar wall thickening in the lungs of the feline in the PBS-GEL 02, CDE, and IV groups, primarily targeting alveolar epithelial cell types (Fig. 6a). A few sporadic positive signals were observed in the lungs of the CDE-GEL 02 and IV-GEL 02 groups. This phenomenon is correlated with the above HE findings and may be associated with the duration of subclinical infection. A small number of scattered positive signals were observed in the larynx and trachea of the feline in the PBS-GEL 02, CDE, and IV groups (shown by red and blue arrows, respectively), and shedding of the mucosal layer was also observed. The IV-GEL 02 group was similar to the CDE-GEL 02 group, with almost no detectable positive signals in the larynx or trachea and an intact, smooth mucosal layer.

The PBS-GEL 02, CDE and IV groups had copies (10^3^–10^6^/ml) of viral genomic RNA in the ENM swab samples after inoculation. In contrast, the viral loads were decreased significantly in the CDE-GEL 02 and IV-GEL 02 groups (Fig. 6b). The virus copies number of the CDE-GEL 02 and IV-GEL 02 groups was increased at 3–8 days post inoculation (dpi) and decreased continuously for 10–21 dpi. The apparent reduction in viral loads in the CDE-GEL 02 group indicates the great potential of CDE-GEL 02 as a vaccine for the prevention of FCV disease.

## Discussion

FCV infection is common in domestic cats and can cause acute upper respiratory illness [1]. Highly virulent strains of FCV can cause systemic disease with a high mortality rate [2]. Both FCV and human Noroviruses (NoVs) belong to the Caliciviridae family. NoVs are the most important viral pathogens causing acute gastroenteritis epidemics worldwide and affecting people of all ages. As such, NoVs are a threat to public health, and developing an effective NoV vaccine has been a top priority for preventing viral gastroenteritis [32]. However, the inability to grow NoVs in cell culture is a major challenge in the development of NoV vaccines [33]. Therefore, the development of NoV vaccines and the establishment of animal models have been difficult [34]. Notably, FCV is one of the few Caliciviridae viruses that can be cultured in vitro and produce lesions [34]. Perhaps the potential vaccine model for FCV could provide a reference for the study of NoV vaccines. Because of the dangers of FCV, it is highly recommended that all cats be vaccinated [35]. However, the available live attenuated vaccines for FCV face safety concerns such as virus shedding [36] and reemergence of virulence [20]. Inactivated vaccines have problems such as limited induction of immune responses (focusing on the humoral immune response) [20] and incomplete inactivation [19]. Subunit vaccines have efficient antigens from *E. coli* expression systems and thus have the advantages of high production and safety [37]. Therefore, the immunogenicity of the CDE region containing the dominant antigenic epitope of FCV was evaluated. In addition to humoral immunity, cellular immunity was induced in the CDE-GEL 02 group, and this immunity was superior to that induced by the inactivated vaccine (Fig. 4). Due to the antigen characteristics and size of subunit vaccines, they can be more efficiently taken up and presented by antigen-presenting cells, thereby stimulating humoral and cellular immunity and the production of Th1 cytokines [38, 39]. Studies have shown that short peptides with 8 to 10 amino acids truly activate T cells [21]. We considered that the small but precise CDE protein would be able to display the dominant epitopes more accurately, facilitating the recognition and presentation of APCs and therefore activating T-cell immunity more effectively. There are few reports on FCV subunit vaccines, and the immunogenicity of VLPs formed by VP1 has not been thoroughly studied [4, 6]. The cost, expression quantity, time period and protein stability of baculovirus expression used in VLPs research are also major problems [23–25]. Short CDE peptides containing effective FCV epitopes have significant advantages in terms of time, cost and expression. Here, for the first time, we comprehensively evaluated the immunogenicity and efficacy of the CDE region of the FCV VP1 protein in eliciting humoral, mucosal and cellular immunity. The CDE subunit vaccine induced an effective and balanced immune response.

In terms of humoral immunity, the CDE-GEL 02 group exhibited significant increases in the serum IgG, IgA, and NAbs level, which is consistent with the good immunogenicity of the FCV VLPs subunit vaccine reported in the literature [4]. However, the level of humoral immunity induced by the inactivated vaccine was greater than that induced by CDE-GEL 02. This finding is similar to the fact that the commercial inactivated FCV vaccine can produce significant humoral immune results [27]. The reason why the ability of CDE protein to stimulate humoral immunity is weaker than that of the inactivated vaccine may be the smaller molecular weight of CDE. A few short peptides can induce T-cell immune activity but usually have lower immunogenicity [21]. The molecular weight of CDE is only 14.8 kDa, and CDE can induce only a slightly weak humoral immune response. To further improve the efficacy of the CDE protein in humoral immunity, we may attempt to express the CDE protein in tandem in future studies. The strategy of tandem expression may improve immunogenicity due to the increase in the overall protein molecular weight. Studies of NoV studies have shown that both the P particle and the polyvalent P domain complexes of VP1 induced significantly higher antibody and CD4+ T-cell responses in mice than those induced by free P dimers [40]. In a study of the COVID-19 vaccine, tandem expression of the receptor binding domain (RBD) dimer as a subunit antigen increased the stability of the antigen and showed good safety and immunogenicity in clinical trials [41]. Alternatively, fusion expression of the CDE protein with Salmonella flagellin (adjuvant carrier effect) could be attempted to further improve its immunogenicity [42]. Surprisingly, CDE-GEL 02 was more effective at inducing mucosal immunity. In particular, the production of IgG and NAbs in swab samples was comparable to that obtained with the inactivated vaccine. Swab IgA production in the CDE-GEL 02 group was also significantly higher than that in the PBS-GEL 02 group. We thought that the reason why the CDE protein can produce effective mucosal immunity may be the addition of nasal drops to the immunization program. It has been reported that nasal administration of vaccines plays an important role in inducing mucosal and systemic immune protection [43]. For SARS-CoV-2, the importance of nasal drop immunization in stimulating the mucosal immune system has been shown [44]. Oral fauces are the primary site of persistent FCV replication [1]. During mucosal viral infection, local NAbs and sIgA induced by the vaccine act as the first line of defence. Pathogens are destroyed by mucosal NAbs and sIgA upon first invasion, thereby preventing subsequent infection [44]. Several studies have demonstrated the efficacy of combined subcutaneous and intranasal immunization with feline herpesvirus, another major respiratory pathogen [45–47]. After systemic immunity is established by subcutaneous immunization, an intranasal booster immunization is administered to enhance local mucosal immunity [47]. The limitation of this study is that we did not further explore the best immune program for accessing the CDE subunit vaccine. We did not explore the specific effects of different combinations of subcutaneous and nasal immunization or the sequence of immunization on the immune effect of the CDE protein. It is possible that different immunization regimens may have some influence on the immune effect. However, it is clear that the CDE-GEL 02 with combined immunization can produce an effective immune effect. In the CDE and IV groups lacking the GEL 02 adjuvant, particularly weak antibody levels were produced after immunization. This finding also demonstrates the importance of adjuvants for the immunogenicity of subunit vaccines and inactivated vaccines [48].

The cytokine TNF-α is produced by CD4+ Th1 cells and powerfully stimulates the development of a Th1 cell response [49]. We found that the expression of TNF-α was upregulated after immunization with CDE-GEL 02 and that IV-GEL 02 had the opposite effect. Moreover, the proportion of CD4+ T lymphocytes increased in both groups. In view of these findings, CD4+ T cells can be divided into helper Th1 cells and Th2 cells, and these two cell populations are mutually inhibitory [50–53]. The Th1 cells secrete related cytokines to promote cellular immunity while inhibiting humoral immunity. The Th2 population is involved in humoral immunity, and when this population is dominant, it inhibits cellular immunity. Therefore, we hypothesize that CDE-GEL 02 may preferentially promote the proliferation of Th1 cells and increase the level of the Th1-type cytokine TNF-α. The IV-GEL 02 may promote the proliferation of Th2 cells and promote the dominance of humoral immunity (the results in the manuscript also show that the IV-GEL 02 group induces better humoral immunity than the other groups), while inhibiting the Th1 cell population and the levels of related cytokines, leading to the decrease in the TNF-α levels. The TNF-α levels reflect the strength of cellular immunity to some extent. TNF-α can activate various cell functions, such as activating APCs, CD4+ and CD8+ T cells and recruiting NK cells [54]. Therefore, our results suggest that CDE-GEL 02 can effectively stimulate CD4+ T lymphocyte immunity and increase the level of the effector TNF-α.

We performed a challenge experiment after immunization and found that both the CDE-GEL 02 and IV-GEL 02 groups prevented severe disease but were not protected from FCV infection, which is a general deficiency in the immune efficacy of FCV vaccines [16–18]. The deficiency of this study is the lack of a comparison of the immune effects of the CDE protein with those of the VP1 protein. This approach may more intuitively highlight the immunogenicity of CDE and thus determine whether the CDE protein is suitable for replacing VP1 as an effective subunit vaccine against FCV. The E region is highly variable [9, 11, 12]. This may also explain the poor cross-protection ability of the FCV vaccine against heterologous strains in the clinic [4, 12]. Although the E region of the FCV is variable, the CD region is a relatively conserved fragment [9], and its function as a support carrier to stabilize the E protein has not changed. Therefore, this study can provide a reference model for other heterologous FCV vaccines and polyvalent vaccines.

## Conclusions

In summary, the prediction of the epitopes of FCV VP1 in this study found that the dominant antigen region was located in the E region. The conformationally stable CDE protein was used as an immunogen. The immunogenicity and efficacy of the FCV VP1-truncated CDE protein were evaluated comprehensively in terms of humoral, mucosal and cellular immunity for the first time. The CDE subunit vaccine with the adjuvant GEL 02 could induce an effective balanced immune response. A challenge trial showed that the protection generated by the CDE-GEL 02 subunit vaccine significantly reduced the severity of disease in animals. This study indicated that the CDE protein could be a promising candidate subunit vaccine for preventing FCV infection in felines.

## Methods

### Cell lines and virus

Crandell Reese Feline Kidney (CRFK) cells were obtained from the China Center for Type Culture Collection (Wuhan, China) and cultured in Dulbecco’s modified Eagle’s medium (DMEM, Gibco, Waltham, MA, USA) supplemented with 10% foetal bovine serum (Invitrogen, CA, USA) and 1% penicillin–streptomycin solution (Beyotime, Wuhan, China) at 37 °C and 5% CO_2_ in a humidified incubator. FCV strain W was isolated and identified in our laboratory (GenBank: OQ673104). The strain was cultured for CDE gene cloning for use as both an IV and challenge strain. CRFK cells were inoculated at a density of 80–90% with 0.01 MOI of FCV strain W. Twenty-four hours after inoculation, FCV produced cytopathic effects (CPEs), such as cell shrinkage, rounding, and drift. The samples were centrifuged to harvest the supernatant. The titre of FCV was determined by the following methods. A sample (100 μL) of virus was diluted at a 10^−1^–10^−10^ ratio, added to a 96-well plate of CRFK cells (100 μL of 1 × 10^5^/mL), and cocultured for 4 days at 37 °C and 5% CO_2_. The number of cytopathic pores in each dilution was calculated using the Reed and Muench method and recorded.

### Obtaining the sequence of the VP1 gene of FCV strain W

A FastPure Viral DNA/RNA Mini Kit (Vazyme, RC311, China) was used to extract RNA from FCV strain W according to the manufacturer’s instructions. cDNA was obtained using Vazyme’s RT–PCR enzyme. cDNA was used as a template for amplification of the VP1 gene with the following primers: forward, 5′-ATGTGCTCAACCTGCG-3′, and reverse, 5′-TCATAATTTAGTCATGCTACTCC-3′. PCR was carried out using Phanta Super-Fidelity DNA Polymerase (Vazyme, P501-d1, China) in a thermocycler (Eastwin Life Sciences, ETC811, China). The VP1 PCR products were identified by agarose gel electrophoresis, the band size was 2007 bp, and the products were subsequently sent to Tsingke Biotechnology Company for sequencing.

### Prediction of the VP1 protein structure with AlphaFold2

The obtained FCV strain W VP1 amino acid sequence (669 amino acid residues) was uploaded to the AlphaFold2 website (alphafold.ebi.ac.uk/) for protein structure prediction. The AlphaFold prediction process primarily consists of five steps: multiple sequence alignment (MSA) construction, template search, inference with five models, model ranking based on the mean pLDDT and constrained relaxation of the predicted structure [55]. The highest-ranking model among the five models was used in a follow-up test (FCV W VP1 ranked-0.pdb). The mean median pLDDT of this model was 86.61829885120392. PyMOL software was used to compare the prediction model file with the FCV VP1 4 PB6 file uploaded to the Protein Data Bank (PDB DOI: 10.2210/pdb4PB6/pdb), and the average root mean square deviation (RMSD) was 3.781.

### Antigen epitope prediction

The predicted FCV strain W VP1 protein structure PDB file was uploaded to the Spatial Epitope Prediction of Protein Antigens 3.0 website (SEPPA 3.0 badd-cao.net) for the prediction of epitopes. The following settings were used: upload PDB file, A chain, unspecified antigen protein subcellular localization, species of host, 0.19 threshold, and enter email address. The forecast results are presented in a .txt file. These included the sequence position of amino acids, amino acid names, the score of each amino acid, and the spatial position of amino acid residues on the structure (surface or core). The dominant epitope region was identified based on the score (Table 1).

### Cloning, expressing, purifying and identifying the CDE protein

For the FCV W cDNA template, amplification of the CDE gene was performed with the following primers: forward, 5′-AAGGAGATATACATATGAAGGATGGGGCAA-3′, and reverse, 5′-GTGGTGGTGGTGGTGGTGCTCGAGATCAGATGTTTGCAC-3′. PCR was carried out using Phanta Polymerase in a thermocycler. The CDE PCR products were identified by agarose gel electrophoresis. These products were homologously recombined with pET-42b (NdeI/XhoI), cut by a linearization enzyme and subsequently transformed into *E. coli* DH5α receptor cells. After the bacterial solution was identified correctly, it was sent to the Tsingke Biotechnology Company for sequencing. The recombinant expression plasmid pET-42b-CDE was extracted after correct sequencing.

The plasmid pET-42b-CDE was transformed into *E. coli* BL21 (DE3) cells for expression. The recombinant strain BL21-pET-42b-CDE was cultured in lysogeny broth (LB) medium supplemented with 50 μg/mL kanamycin at 37 °C until the optical density at 600 nm (OD600) reached 0.6–0.8, and the cells were then induced with 1.5 mM IPTG and incubated with shaking for 16 hours at 18 °C. A blank vector was used as a negative control.

For protein purification, 2 L of culture was pelleted by centrifugation at 7100 g (10 min at 4 °C), resuspended in phosphate-buffered saline (PBS) and lysed using an ultrahigh-pressure cell disrupter (ATS Engineering, Inc.). After centrifugation, the supernatant was filtered through a 0.22-μm-pore-size filter and loaded onto a HisTrap HP column (GE Healthcare). The target protein was eluted with a linear gradient of binding buffer A (20 mM Tris-HCl and 500 mM NaCl [pH 7.4]) and elution buffer B1 (20 mM Tris-HCl, 500 mM NaCl, and 500 mM imidazole [pH 7.4]). The protein was further purified via SEC using a Superdex200 gel filtration column (GE Healthcare) equilibrated with buffer B2 (20 mM Tris-HCl and 200 mM NaCl [pH 7.4]). To increase the concentration such that the solution could be preserved, the purified protein was concentrated to 40 mg/mL using a 3-kDa molecular mass cut-off (Millipore, UFC900396, USA) centrifuge concentrator, and the concentration was determined by measuring the absorbance at 280 nm with a NanoDrop spectrophotometer (Thermo Scientific). The protein sample was flash-frozen with liquid nitrogen and stored at − 80 °C.

The protein samples were treated with sodium dodecyl sulfate (SDS) loading buffer and boiled for 10 min. The proteins were separated by 12% SDS–PAGE and then stained with Coomassie brilliant blue solution. The proteins were subsequently transferred onto PVDF membranes (BIO-RAD, 1620177, USA). The membranes were blocked with 5% skim milk for 3 h at room temperature and then incubated with a 1:5000 dilution of HRP-conjugated 6*His-Tag mouse IgG monoclonal antibody (Proteintech, HRP-66005, USA) overnight at 4 °C. The protein bands were visualized using BeyoECL Plus Western ECL Blotting Substrate (Beyotime, P0018S, China).

### Vaccine preparation

The vaccines included CDE-GEL 02, FCV W-IV-GEL 02, PBS-GEL 02, CDE and IV. For the CDE-GEL 02 vaccine, 100 μg of preserved CDE protein was diluted to 900 μL with PBS. Mixing was performed with MONTANIDE™ GEL 02 aqueous adjuvant (Seppic, France) at a volume ratio of 9:1, 100 μg and 1 mL per cat, with vortexing for 10 min. For the FCV W-IV-GEL 02, viruses were added to β-propanolide (Macklin, 57–57-8, China) at 1:1000, inactivated for 24 h at 4 °C, and then hydrolysed at 37 °C for 2 h to make the hydrolysis of β-propanolide completely carcinogenic. After complete inactivation, the virus amount of 10^8^ TCID_50_ was matched with GEL 02 water adjuvant according to a volume ratio of 9:1 (1 mL per cat), and the mixture was mixed for 10 min. For PBS-GEL 02, 900 μL of PBS was mixed with 100 μL of GEL 02 adjuvant as described above. For CDE and IV, 100 μg of CDE protein and 10^8^ TCID_50_ of inactivated FCV strain W were mixed with PBS, respectively.

### Immunization, challenge and sample collection

The immunogenicity of the CDE protein vaccine was evaluated in rural felines aged between 8 and 12 weeks; the results of FCV, feline herpesvirus, feline panleukopenia virus antigen and antibody tests were negative. The felines were purchased from Jiaxing Huarong Cat Farm, Shandong Province, China, and randomly divided into 6 groups: CDE-GEL 02 group (*n* = 6), IV-GEL 02 group (*n* = 3), PBS-GEL 02 group (*n* = 3), CDE group (*n* = 3), IV group (*n* = 3) and mock group (*n* = 3). The five groups were immunized with 100 μg of CDE protein with GEL 02, 1 ml of IV with GEL 02, 1 ml of PBS with GEL 02, 100 μg of CDE protein and 1 ml of IV (Table 2). The IV-GEL 02 and IV groups were treated following the commercial immunization strategy of subcutaneous injections at 0 W, 2 W and 5 W. To stimulate the corresponding mucosal immunity, the CDE-GEL 02, CDE and PBS-GEL 02 groups underwent additional nasal drop immunization. Subcutaneous injection was given at 0 W and 5 W, and nasal immunization was given at 2 W. Serum and ENM swabs were collected at 0 W, 5 W and 7 W after immunization and were used to detect IgG, IgA, NAbs, TNF-α and T lymphocytes. The collected blood was incubated at 37 °C for 1 h such that the serum fully precipitated. The sample was centrifuged at 5000 g for 10 min at room temperature. The supernatant was removed, and part of the serum was inactivated at 56 °C for 30 min for the detection of antibodies. The other part was directly stored at − 20 °C without inactivation for cytokine detection. The ENM swabs were centrifuged at 10000 g for 10 min at 4 °C, and the supernatant was filtered through a 0.22 μm filter and stored at − 20 °C for the detection of antibodies. FCV strain W, a 10^7^ TCID_50_/mL challenge dose per feline, was administered to each immune group at the 7th week after immunization. Previous studies in our laboratory have shown that FCV strain W can cause obvious FCV-related symptoms.

**Table 2:** Immunization program and grouping

Group	Antigen	Dose	Adjuvant	Route	Schedule
**CDE-GEL 02**	**CDE**	**100 μg**	**GEL 02**	**SC + IN + SC**	**0 W: Once** **2 W: Twice** **5 W: Thrice**
**IV-GEL 02**	**IV**	**10**^**8**^ **TCID**_**50**_		**SC + SC + SC**	
**PBS-GEL 02**	**–**	**1 mL**		**SC + IN + SC**	
**CDE**	**CDE**	**100 μg**	**–**	**SC + IN + SC**	
**IV**	**IV**	**10**^**8**^ **TCID**_**50**_	**–**	**SC + SC + SC**	

### Determination of IgG and IgA antibody levels

The levels of IgG and IgA in serum and swabs were measured by ELISA. Polystyrene microlitre 96-well plates were coated with 100 μL of 1 μg/mL CDE protein in coating buffer (15 mM Na_2_CO_3_, 35 mM NaHCO_3_, 3 mM NaNO_3_, pH = 9.6) for 2 h at 37 °C. After 2 h of blocking with 1% BSA at 37 °C, the collected samples were serially diluted in PBST, added in triplicate, and incubated at 37 °C for 1 h. Then, an HRP-conjugated goat anti-cat IgG or IgA antibody (BETHYL, ARG21743.500, A20-101P, China) was added to each well (1:5000), and the mixture was incubated for 45 min at 37 °C. The plates were washed 5 times during each step. Finally, 50 μL of chromogenic solutions A and B were added to each well. 50 μL of stop solution was added after 10 mins. The OD values at 630 nm and 450 nm were measured using a microplate reader (Thermo Fisher Scientific).

### Viral neutralization assays

Serum and swabs samples collected from immunized felines were serially diluted with cell culture medium at a twofold ratio. The diluted sera were mixed with a viral suspension of 100 TCID_50_ in 96-well plates at a ratio of 1:1, followed by 2 hours of incubation at 37 °C in a 5% CO_2_ incubator. CRFK cells were then added to the serum-virus mixture, and the plates were incubated for 4 days at 37 °C in a 5% CO_2_ incubator. The CPE of each well was recorded via microscopic observation, and the neutralizing titre was calculated by the dilution number of the 50% protective condition.

### Cytokine detection

The levels of secreted TNF-α were determined using commercial ELISA kits (Fankew, F68007-A, China) according to the manufacturer’s recommendations. Cytokine was quantified from the standard curves prepared from standard reagents provided by the kits, and the OD value was detected at 450 nm for each plate using a microplate reader.

### Flow cytometry

Seven weeks after immunization, blood from each group was collected into anticoagulant tubes containing heparin sodium, 20 times the volume of whole-blood red blood cell lysis buffer (BioLegend, 420,301, USA) was added, and the samples were lysed for 10 min at room temperature. After lysis, the cells were centrifuged at 400 g for 5 min at 4 °C. Leukocytes were resuspended in PBS and counted. Then, 0.2 μg of mouse anti-feline CD4-PE and CD8-FITC antibodies (SouthernBiotech, 8130–09, 8120-02, USA) were added to each sample of 10^6^ cells, and the resulting mixture was incubated at 4 °C in the dark for 30 min and washed twice with PBS. Surface antigen fixation and membrane permeation of CD4+ and CD8+ cells were performed by adding fixation buffer (BioLegend, 420,801, USA) and intracellular staining permeabilization wash buffer (BioLegend, 421,002, USA). The anti-CD3–647 (Bio-Rad, MCA1477T, USA) antibody was added, and the samples were incubated for 30 min at 4 °C in the dark and washed twice with wash buffer. The cells were resuspended in 500 μl of buffer and filtered through a 70-μm cell mesh sieve. Flow cytometry (Beckman Coulter, Cytoflex, USA) was performed, and the results were analysed with FlowJo software.

### Clinical evaluation

Felines were monitored at room temperature in a clean, one-cat, one-cage environment. Felines were observed daily for 21 days post challenge. A previously designed clinical symptoms FCV scoring system was implemented [56]. The clinical observers assess the clinical score parameters of each animal, including body temperature, weight loss rate, mental state, and ENM symptoms at the same time each day. Daily clinical scores were calculated by summing the numerical values of these clinical parameters.

### RT–PCR

ENM swabs were collected daily after viral challenge and used for RT–PCR assays. Total RNA was extracted from swabs as described previously with a FastPure Viral DNA/RNA Mini Kit and ChamQ Universal SYBR qPCR Master Mix (Vazyme, Q711–02, China). The forward and reverse primers targeting the FCV gene used for RT–PCR were 5′-GACAATGTCTCAAACTCTGAGCTTCGT-3′ and 5′-GTGAGCTGTTCTTTGCACAT-3′, respectively. RT–PCR was performed under the following reaction conditions: 95 °C for 30 s, followed by 40 cycles of 95 °C for 10 s, 60 °C for 30 s, 95 °C for 15 s, 60 °C for 60 s and 95 °C for 15 s.

### Histopathological observation

One feline from each group were euthanized 21 days after challenge by intravenous injection of sodium pentobarbital overdose (100 mg to 200 mg/kg). The lungs, larynx and trachea were collected and immediately immersed in 4% formalin solution for histopathological assay. The tissues were dehydrated and embedded conventionally to make paraffin slices. HE staining was performed by an automatic staining machine, and histopathological changes were observed by optical microscopy.

### Immunohistochemistry

Paraffin sections were dewaxed in water and treated with 3% hydrogen peroxide for 30 minutes. Antigen recovery was performed by superheat repair in antigen repair buffer. The slides were plugged with normal goat serum (BOSTER, China) and incubated overnight in a humid chamber at 4 °C with anti-FCV antibodies. The stained sections were incubated with enzyme-labelled goat anti-mouse IgG polymer (GeneTech, China) for 30 min. After each incubation step, the sections were washed 3 times with 0.01 M PBS for 5 min. The slices were incubated with 3,3′- DAB solution (Solarbio, China), and antigen localization was observed. The sections were weakly counterstained with haematoxylin. The corresponding homotypes were used as negative controls. The sections were scanned with a Leica pathology scanner.

### Statistical analysis

All data were obtained from at least three independent experiments, and the results are presented as the means ± standard deviations (SDs). The statistical analysis was performed using column analysis t tests and two-way analysis in Graph Pad Prism 8.0 (GraphPad Software, Inc., USA). Various degrees of significance were designated as follows: ^*^ *p* < 0.05, ^*^^*^ *p* < 0.01, ^*^^*^^*^ *p* < 0.001, **** *p* < 0.0001, and ns indicates no significant difference.

### Supplementary Information

**Additional file 1.** Prokaryotic expression of the CDE protein. The expression of recombinant CDE protein was assessed by SDS–PAGE analysis, as shown in Fig. 2a in the manuscript.

**Additional file 2.** Determination of CDE protein expression by Western blotting. Antigenicity was verified using an HRP-conjugated 6*His Tag mouse antibody, as shown in Fig. 2c in the manuscript.

## Data Availability

The datasets used and analysed during the current study are available from the corresponding author upon reasonable request. The DNA sequence GenBank accession number of the VP1 protein is OQ673104.

## Abbreviations

FCV: Feline calicivirus
ORFs: Open reading frames
Pre-VP1: Capsid precursor protein
LC: Leader capsid protein
VLPs: Virus-like particles
NAbs: Neutralizing antibodies
sIgA: Secretory IgA
aa: Amino acids
Ags: Antigenic sites
SEC: Size-exclusion chromatography
IV: Inactivated vaccine
ENM swabs: Eye, nose and mouth swabs
NoVs: Human noroviruses
RBD: Receptor binding domain
CRFK: Crandell-reese feline kidney
DMEM: Dulbecco’s modified Eagle’s medium
SEPPA 3.0: Spatial epitope prediction of protein antigens 3.0
SC: Subcutaneous
IN: Nasal drop immunization
