# Supplementary figures and images for: The CDE region of feline Calicivirus VP1 protein is a potential candidate subunit vaccine
Source: BMC Vet Res. 2024 Mar 5;20:80. doi: 10.1186/s12917-024-03914-2 (PMC10916247; doi:10.1186/s12917-024-03914-2)

## Slide 1
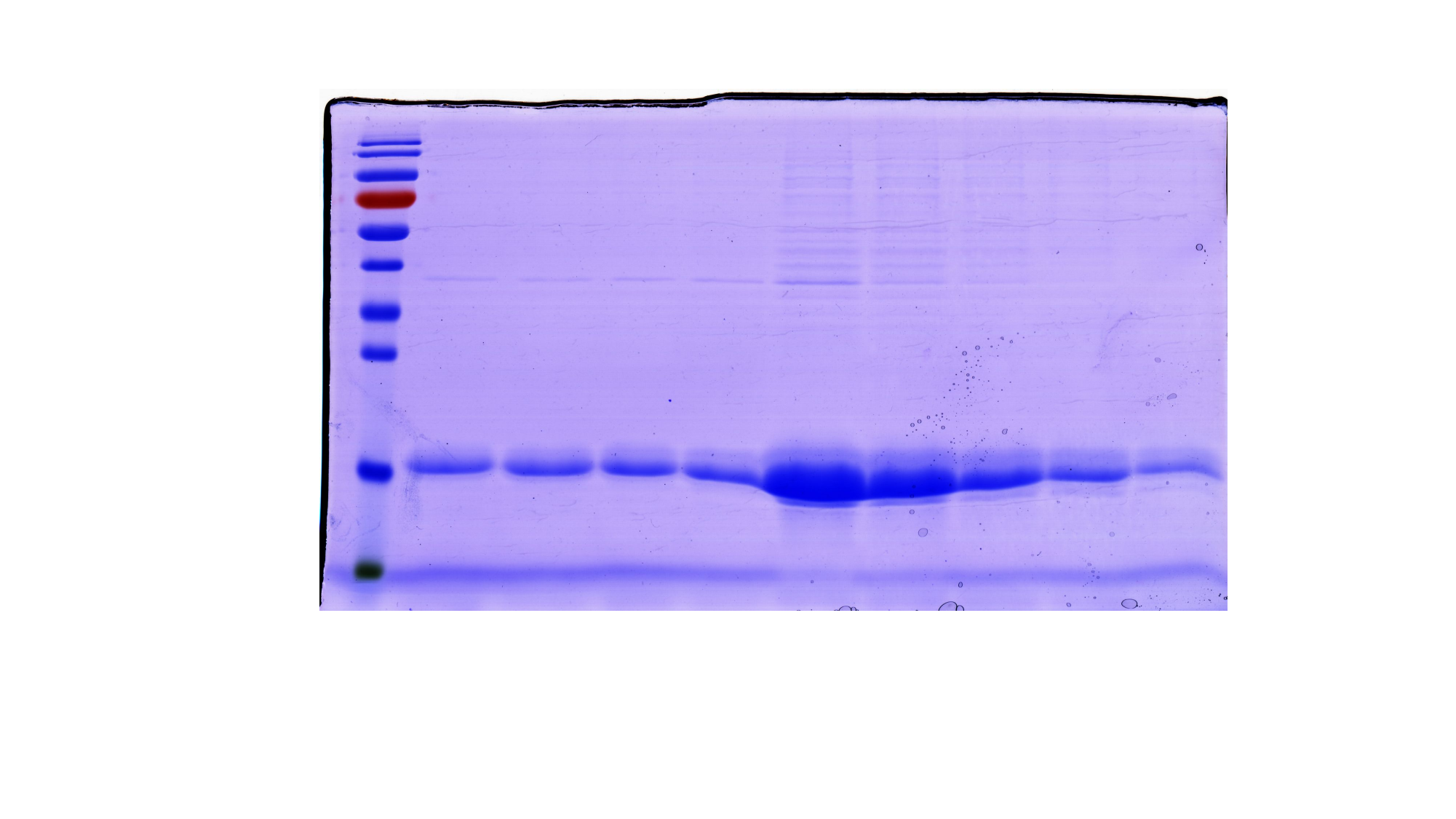

Supplement: Supplementary file 1 — Additional file 1. Prokaryotic expression of the CDE protein. The expression of recombinant CDE protein was assessed by SDS–PAGE analysis, as shown in Fig. 2a in the manuscript. [file 12917_2024_3914_MOESM1_ESM.pptx]

## Slide 1
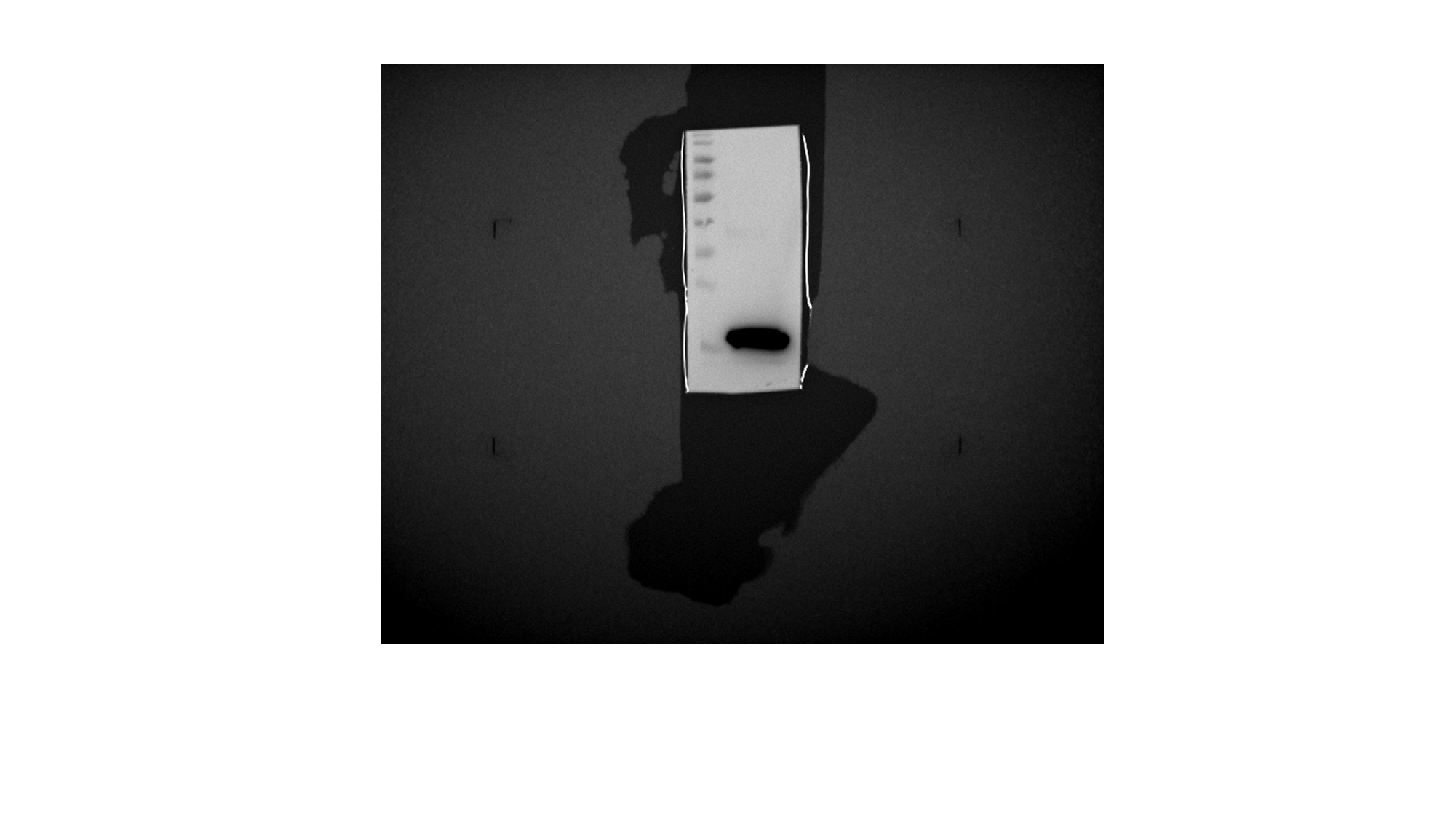

Supplement: Supplementary file 2 — Additional file 2. Determination of CDE protein expression by Western blotting. Antigenicity was verified using an HRP-conjugated 6*His Tag mouse antibody, as shown in Fig. 2c in the manuscript. [file 12917_2024_3914_MOESM2_ESM.pptx]
